# Neuropsychiatric symptoms, quality of life and caregivers’ burden in dementia

**DOI:** 10.1515/med-2020-0124

**Published:** 2020-09-14

**Authors:** Réka Majer, Olar Adeyi, Zsuzsa Bagoly, Viktória Simon, László Csiba, László Kardos, Tibor Hortobágyi, Ede Frecska

**Affiliations:** Department of Psychiatry, Faculty of Medicine, University of Debrecen, Debrecen, Hungary; Department of Neurology, Faculty of Medicine, University of Debrecen, Debrecen, Hungary; MTA-DE Cerebrovascular and Neurodegenerative Research Group, Debrecen, Hungary; Division of Clinical Laboratory Science, Faculty of Medicine, University of Debrecen, Debrecen, Hungary; Department of Psychiatry and Psychotherapy, Faculty of Medicine, Semmelweis University, Budapest, Hungary; Hygiene and Infection Control Services, Kenézy Gyula University Hospital, University of Debrecen, Debrecen, Hungary; Department of Pathology, Faculty of Medicine, University of Szeged, Szeged, Hungary; Department of Old Age Psychiatry, Institute of Psychiatry Psychology & Neuroscience, King’s College London, London, United Kingdom

**Keywords:** caregivers, caregivers’ burden, dementia, illness intrusiveness, neurocognitive disorders, neuropsychiatric symptoms of dementia, quality of life

## Abstract

The objective of this research is to identify the relationship between the neuropsychiatric symptoms (NPSs) of patients with major neurocognitive disorder (mNCD), their quality of life, illness intrusiveness and the caregiver’s burden. We assessed 131 patients with mNCD. Examination methods included WHO well-being index short version, illness intrusiveness rating scale, Alzheimer’s Disease Assessment Scale-Cog, Mini Mental State Examination and neuropsychiatric inventory. The results were analysed using standard statistical tests. In our sample, the prevalence of NPSs is 100%. A significant correlation (*p* < 0.0001) was observed with quality of life and illness intrusiveness. Additionally, a strong relationship was observed between NPSs and the caregiver’s burden (*r* = 0.9). The result is significantly twice as much stronger in comparison to the relationship between NPS and cognitive symptoms (*r* = 0.4). This is the first study in Hungary to assess the impact of NPS on the burden of relatives and quality of life. NPS had twice stronger impact on caregivers’ burden than cognitive decline. However, further studies are needed to assess the sub-syndromes in mNCD in relation to NPS.

## 1 Introduction

Dementia is one of the greatest challenges in the twenty-first century as the main risk factor of dementia is old age itself [1]. The WHO forecasts that two billion of the whole population will be over 60 by 2050. Every year, 8 million new dementia cases are diagnosed worldwide, that is, four cases per second. Up to 25 million new dementia cases are predicted by 2050. The rapid growth of dementia cases requires urgent actions, especially in countries with low or middle income. In 2013, more than 15 million family members provided 17.7 billion hours of care to dementia patients, which valued to more than $200 billion. The illness-related costs are estimated at $604 million yearly which exceeds 1% of the global GDP. As a result, dementia is not only the problem of the patients, their family and their close environment, but it is rather the problem of the whole society with economic and social impacts [2].

Regarding costs, neuropsychiatric symptoms (NPSs) represent the most significant problem more than the cognitive decline alone [3]. These include agitation, aggression, irritability, activity disorders, disinhibition, mood disorder, anxiety, hallucination, delusion, sleeping disturbance and eating disorder. NPS can appear at any stage of the illness with varied frequency and severity. The significance of NPS is that inadequate treatment accelerates the progression of the disease, reduces daily activity, impairs quality of life, increases healthcare attendance and burnout of caregivers, which eventually raises costs and advances admission to an aged care facility [4].

In 2010, Alzheimer’s Disease International launched a roundtable with NPSs in focus and outlined the directions of research with respect to medicine development and other disciplines. According to the literature, syndromes that are considered to be of secondary importance next to the cognitive decline have more negative effect on patients’ quality of life and are the defining elements of patients’ distress, intrusiveness and the outcome of the disease [5].

For the clinician, it is simpler to differentiate and define cognitive symptoms related to dementia and scale their severity than to define and treat the secondary symptoms. This is partly the reason why greater attention is paid to these symptoms by clinicians and scientists. NPS, for many years, were considered to be a neglected field of geriatric psychiatry, although the implementation of a testing and nursing strategy would be absolutely crucial and useful to prevent and treat NPS. It has also become necessary to devise and apply a non-drug approach that makes dementia care easier in everyday life, both for patients and for caregivers [6].

NPSs are of great importance since they greatly increase morbidity, mortality and caregivers’ burden. Balance disorder, falls, agitation and aggression behaviour, eating and sleeping disturbances can be the causes and also the consequences of NPS. Balance disorders and falls are part of the agitated disorders, with a 35–40% frequency. Agitation also includes aggressive behaviour. One part of the aggressive behaviour is the verbal aggression such as shouting, screaming and swearing; and the other part of the physical aggression is a passive resistance, rejection of food and drink, refusal of cooperative behaviour and self-harming behaviour. Rejection of basic needs can also be part of a passive suicidal behaviour. The aggression sometimes manifests as insult or crime; 12–50% of dementia patients suffer from eating disturbances, and an annual 4% weight loss is forecasted. This is an important mortality predictor. The prevalence of sleeping disturbance increases (13–40%) as dementia deteriorates. In the most severe cases, the whole circadian rhythm can cease to exist [7].

A 2016 systematic review [8] highlighted that NPS related to dementia has a determining role in daily living and influences both psychological well-being and quality of life of the family. The review also emphasised that for the caregiver, the personality of dementia patients dies years before the actual physical death since they inevitably lose their identity and fall back into a child’s role. The patient is passive, uninterested, unmotivated and the whole situation enhances aggression. Also, sexual and socio-cultural norms are inhibited, which leads to further exclusion and isolation.

People living with NPS experience isolation, fear, diminished quality of life, increased morbidity and mortality, and their medical expenditure also rises [9].

Our program was to examine the prevalence of NPSs in a domestic sample as well as to study the correlation with quality-of-life indicators and the caregiver’s burden.

The long-term goal of this research is to devise a strategy that will help and support patients suffering from neurocognitive disorders and their family caregivers in the long run. The first step is to design a cross-sectional examination of the national sample to survey NPSs and also to measure the related quality of life, disease intrusiveness and caregiver’s burden.

The hypothesis is that a high prevalence of the NPS can be observed in the Hungarian sample, which will significantly affect the patient’s quality of life. Also, the effects of the NPS will have a significant impact on caregivers’ burden.

## 2 Materials and methods

This study was carried out using a sample of patients with neurocognitive disorder who had not previously received any dementia-related drug (*n* = 131). The survey selected samples from patients who were presented between February 2013 and April 2015. The patients attended the geriatric psychiatry consultation in the Psychiatry Clinic and the dementia specialty consultation (neurology clinic) at the University of Debrecen. These patients were included in the survey of those who attended for the first time and were referred by their GP. Additional criteria were that they lived in their homes with their family members, not in a nursing home or other nursing facility, and a family member with whom they were living accompanied them to the examination.

Exclusion criteria were hearing and/or seeing impairment and chronic depression (i.e. the no history of antidepressive therapy depression) or other psychiatric disease in the anamnesis that would have influenced completion of the tests. The examinations were carried out together with the patient and their caregiver. The necessary information was verbally explained to them before the examination commenced and a consent form was also signed by the patients. This research was performed in accordance with the latest version of the Declaration of Helsinki. The study was also approved by the local ethics committee and all the included subjects submitted a written informed consent.

### 2.1 Measurement tools

The first step comprised the demographic overview and the anamnesis with the family member. The next step involved cognitive, neuropsychological and psychological tests.

The examination of the patients was performed with the caregiver being present. At the beginning of the examination, a discussion of the patient’s anamnesis took place, followed by a survey which assessed the patient’s NPS, quality of life and illness intrusiveness. The patient’s relative was present throughout the examination. Upon completion, the selected patients underwent a thorough neuropsychological examination, which took approximately 45–50 min.

The following tests were implemented:

Mini Mental State Examination (MMSE): the most widely used quick cognitive screening test in the international practice that has been extensively used to assess individuals’ cognitive function. The MMSE score ranges from 0 to 30, with higher scores indicating better cognitive function [10].

This method was used during the clinical assessment in the diagnosis of neurocognitive disorder. However, the MMSE results were not included in the analyses because the Alzheimer’s Disease Assessment Scale-Cog (ADAS-Cog) test seemed to be more adequate in evaluating the cognitive functions of the patients from a wider perspective.

### 2.2 ADAS-Cog test

ADAS-Cog: It is one of the most popular cognitive testing instruments in Alzheimer’s-related clinical diagnostic work and research. It consists of 12 subscales, which are pivotal in assessing and monitoring patients with dementia. Although this test is mainly used in Alzheimer’s patients, it is also commonly exploited in other types of dementia as the tests are suitable for generalisation [11]. The validation of the Hungarian version is by Pákáski et al. [12].

### 2.3 Neuropsychiatric inventory (NPI)

The NPI was developed by Cummings et al. to assess dementia-related behavioural symptoms. The NPI examines 12 symptoms of behavioural and psychological functioning: aberrant motor activity, agitation/aggression, anxiety, apathy, appetite and eating abnormalities, delusions, disinhibition, dysphoria, euphoria, hallucinations, irritability/lability and night-time behavioural disturbances. The NPI can screen for NPS in neurocognitive disorder. The NPI is assessed based on the caregiver’s report [13].

### 2.4 WHO well-being index short version (WBI-5)

The well-being index is among the most widely used measuring instrument. It is a self-reported questionnaire about psychological well-being that is used in clinical and monitoring studies. The WHO’s five-item well-being scale is used to collect information about the person’s general well-being from the previous 2 weeks. A validated Hungarian variation is used [14]. The WBI-5 is a short questionnaire consisting of five simple questions, which tap into the subjective well-being of the responders [15].

### 2.5 Illness intrusiveness rating scale (IIRS)

This is a short questionnaire developed by Devins and his colleagues to measure disease intrusiveness. It contains 13 items and the scale ranges from 1 to 7. Higher scores show greater intrusiveness. The underlying concept is that illnesses limit activities that are important for the individual and it interferes with lifestyle and quality of life – which is called illness intrusiveness [16].

### 2.6 Statistical analysis

Statistical analysis and data management were performed by Stata (StataCorp. 2009, Stata Statistical Software: Release 11, College Station, TX, USA) statistical program. The indicated statistical significance was *p* < 0.05. For data analysis, we applied descriptive statistics to present the main characteristics of the sample, calculating the mean and standard deviations. For inferential analysis, Pearson’s chi-square and Fisher’s exact tests were used to examine the differences between the groups and categorical variables. For the analysis of associations between categorical and continuous variables, either ANOVA or Kruskal–Wallis test was applied depending on the normal versus non-normal distribution of the data. And for correlation analysis, Pearson’s correlation coefficient was used. To define the strength of the correlation, we used Akoglu’s user guide, which provides a recommendation for interpreting the correlation coefficient in psychological articles [17]. No *post hoc* testing was performed.

## 3 Results

We summarised the participants’ demographic data (Table 1). Regarding gender, age, MMSE score, ADAS-Cog level and NPI score, no significant difference (*p* > 0.05) was observed.

**Table 1:** Sociodemographic characteristics of participants (*n*  =  131)

	Frequency (*n* = 131 in total)	Percentage (%) of all patients
Gender		
Male	48	36.6
Female	83	63.3
Age		
50–59	7	5.1
60–69	16	12.2
70–79	42	32
80–89	62	47.3
90–99	4	3
Ethnicity		
Caucasian	131	100
Marital status		
Single	6	4.5
Married	61	46.5
Divorced	4	3
Widow	60	45.8
Education		
Grades 1–6	15	11.4
Grades 7–12	42	32
Graduation	45	33.3
Technic	18	13.7
College	7	5.3
University	4	3

	Mean	SD
Age	77	8.3
MMSE	19	5.6
ADAS-Cog	39.4	14.1
NPI total score	44	22.7

In our previous publication, the relationship between cognitive function and NPS was analysed in detail [18]. Therefore, this study’s focus is particularly on the relationship between NPS and quality of life, illness intrusiveness and caregiver’s burden.

### 3.1 Results of NPS assessment

**Figure 1:**
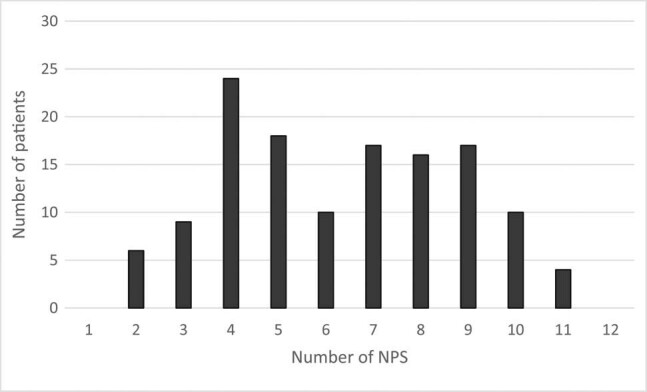
Frequency of NPSs.

In our sample, the occurrence of NPS was 100%, and all patients showed NPS. The minimum number of symptoms was 2, and the maximum was 11. The most common number of symptoms was four, as shown in Figure 1.

The NPI scale was used for NPS measurement. The mean NPI total score was 44.0 (SD = 22.7; range 11–103). With respect to individual symptoms, based on frequency scores, abnormal motor behaviours (2.7 [SD = 1.2]), depression (1.8 [SD = 1.2]), agitation (1.7 [SD = 1.2]) and eating-appetite change (1.4 [SD = 1.3]) were the most frequent in the total sample. The relationship between cognitive function and NPS is described in detail in our previously published article, “Behavioural and psychological symptoms in neurocognitive disorders – specific patterns in dementia subtypes” [18].

### 3.2 Results of the assessment of life quality and NPSs

**Figure 2:**
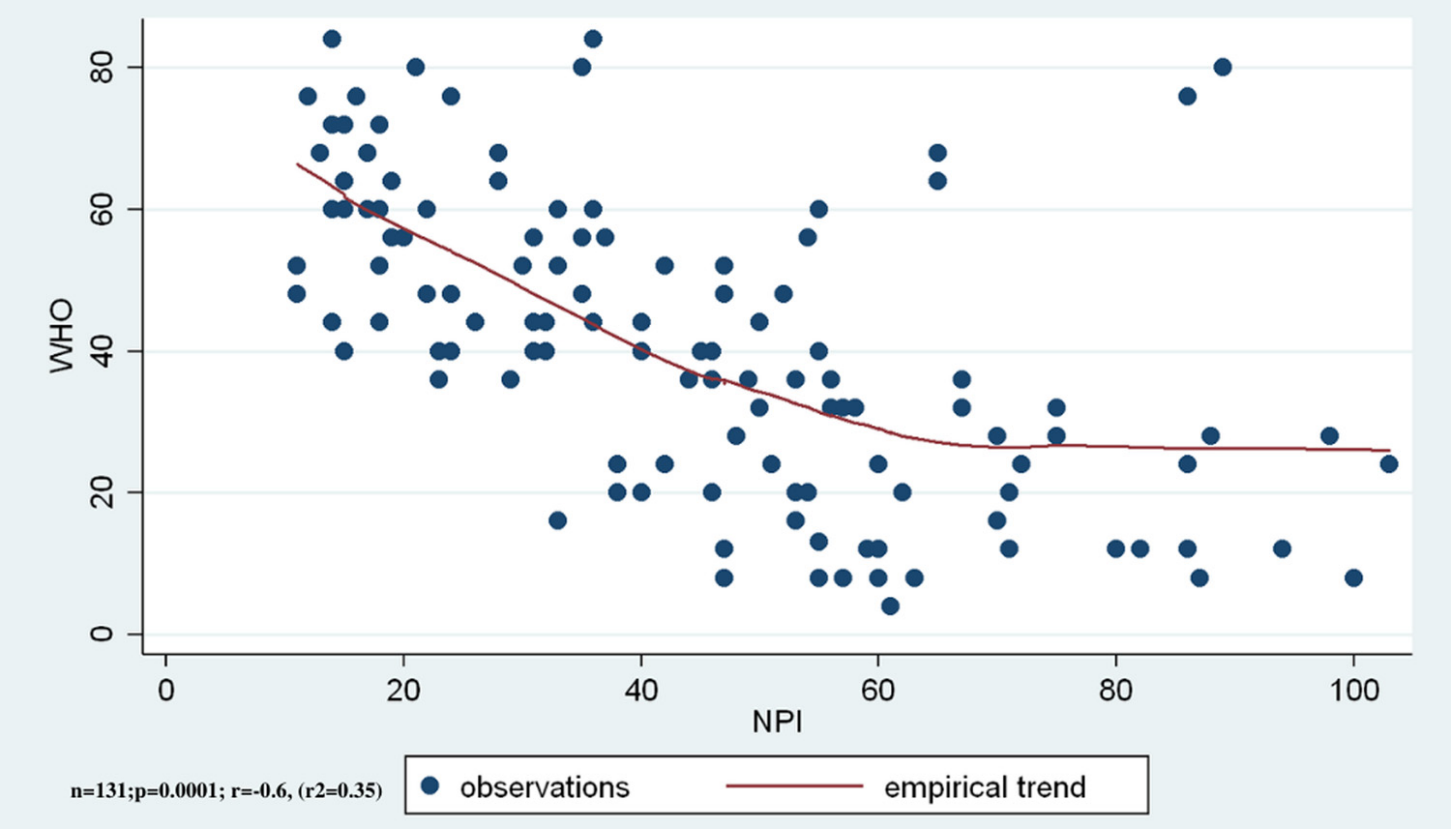
The quality of life is significantly negatively correlated to the NPSs’ total score.

The average WBI-5 was 40.5 ± 21.1. Quality of life shows a pronouncedly significant relation with NPI total score (*r* = −0.6, *r* ^2^ = 0.35, ANOVA *F* (1,129) = 71.62, *p* < 0.0001; Figure 2) as well as with severity and frequency. In all cases, *p* < 0.001 was detected; and in accordance with NPS deterioration, the patient’s quality of life declines.

### 3.3 Results of the assessment of illness intrusiveness and NPSs

**Figure 3:**
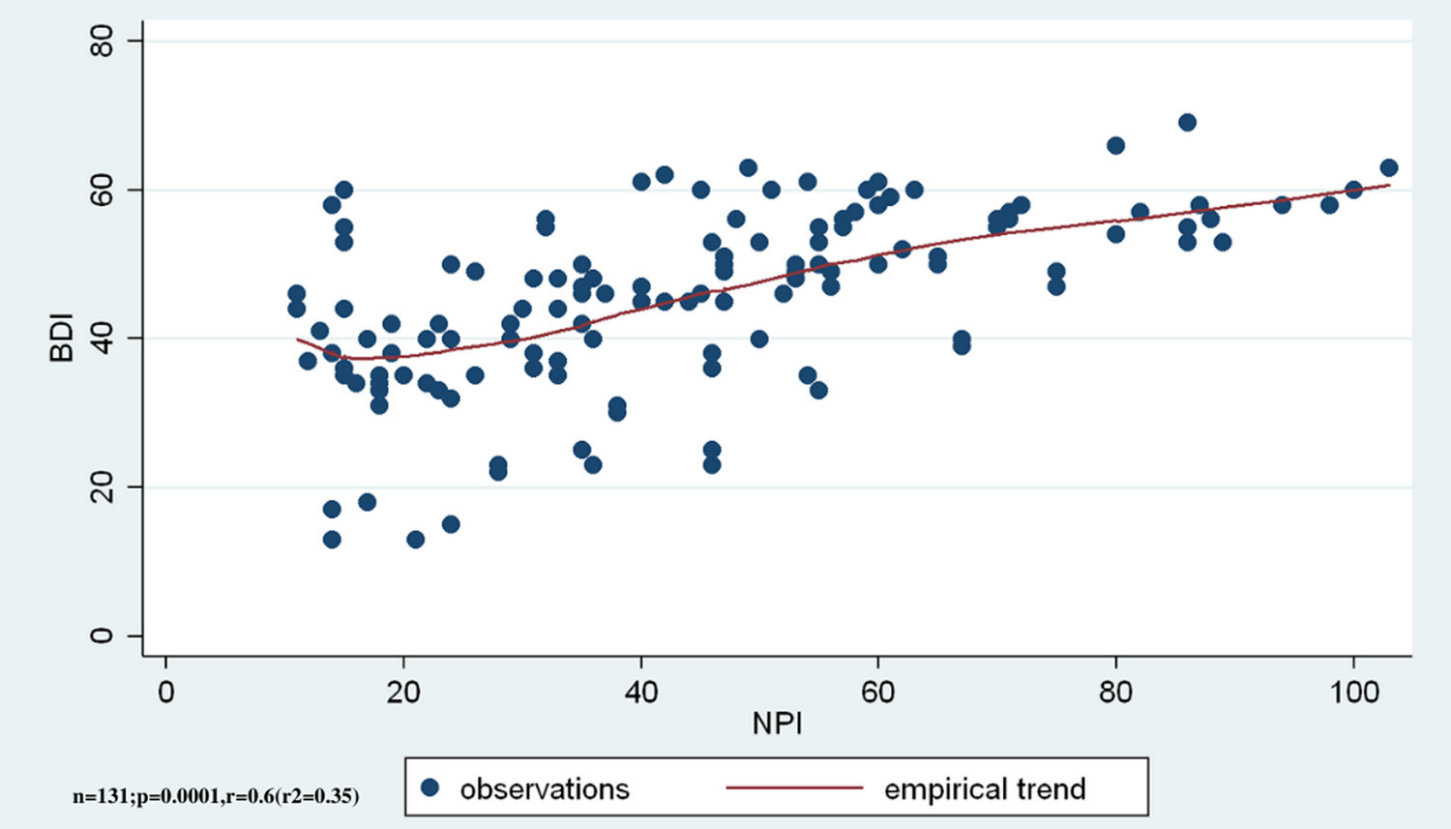
Illness intrusiveness shows significant positive correlation to the total score of NPSs.

The average IIRS was 45.4 ± 11.9. Illness intrusiveness shows significant increase in relation to the total NPI score (*r* = 0.6, *r* ^2^ = 0.35, ANOVA *F*(1,129) = 70.07, *p* < 0.0001; Figure 3). An increase in NPS was associated with an increase in illness intrusiveness.

### 3.4 Results showing the assessment of the caregiver’s distress and NPSs

**Figure 4:**
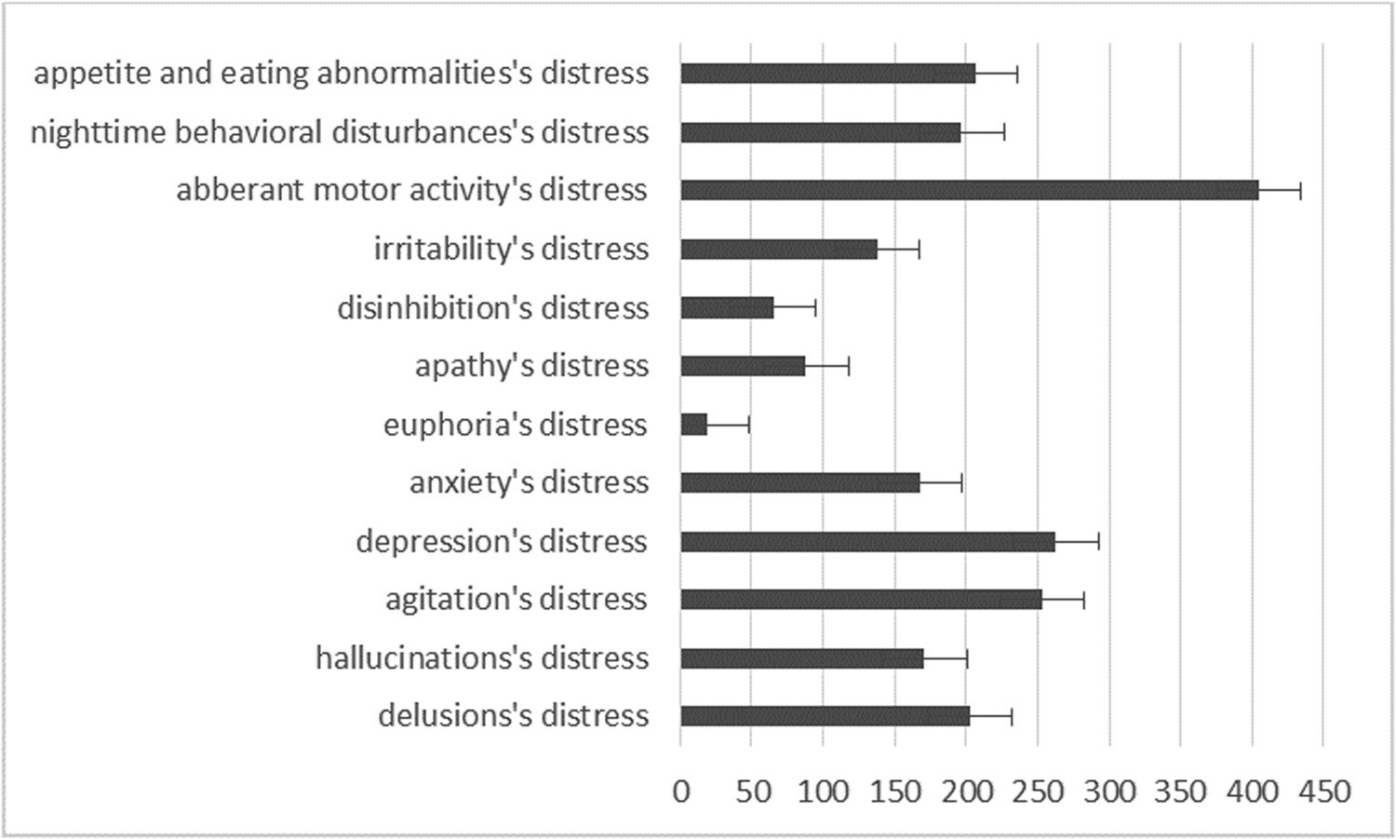
The caregiver’s distress level in the distress factor of neuropsychiatric inventory test.

Here the level of caregiver’s distress is measured using the caregiver’s distress index of NPI test known as “distress factor.” The highest scores were given for aberrant motor behaviour, depression, agitation, eating and appetite changes, delusions and night-time behavioural disturbance (Figure 4).

**Figure 5:**
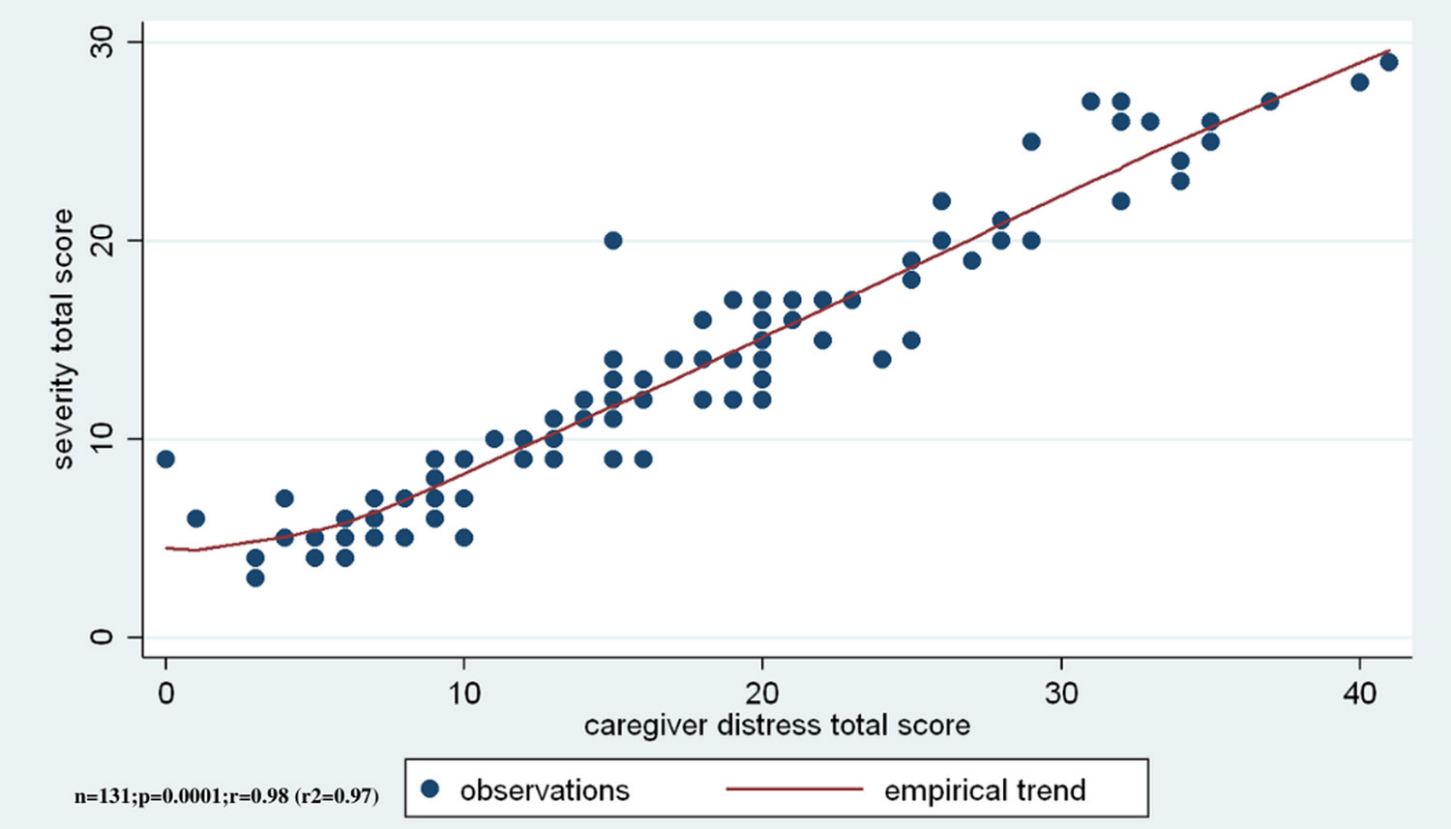
The caregiver’s distress level shows a significant positive correlation to the total score of “NPSs severity.”

In addition, the relationship between the caregiver’s distress index and the total score of NPS as well as the severity and frequency is examined. The three comparisons showed significantly strong correlations (*r* = 0.98, *r* ^2^ = 0.97, ANOVA *F*(1,129) = 1531.02, *p* < 0.001), where the more frequent NPS was associated with the most severe distress of caregiver’s (Figure 5).

### 3.5 The relationship between the caregiver’s burden and cognitive functions

**Figure 6:**
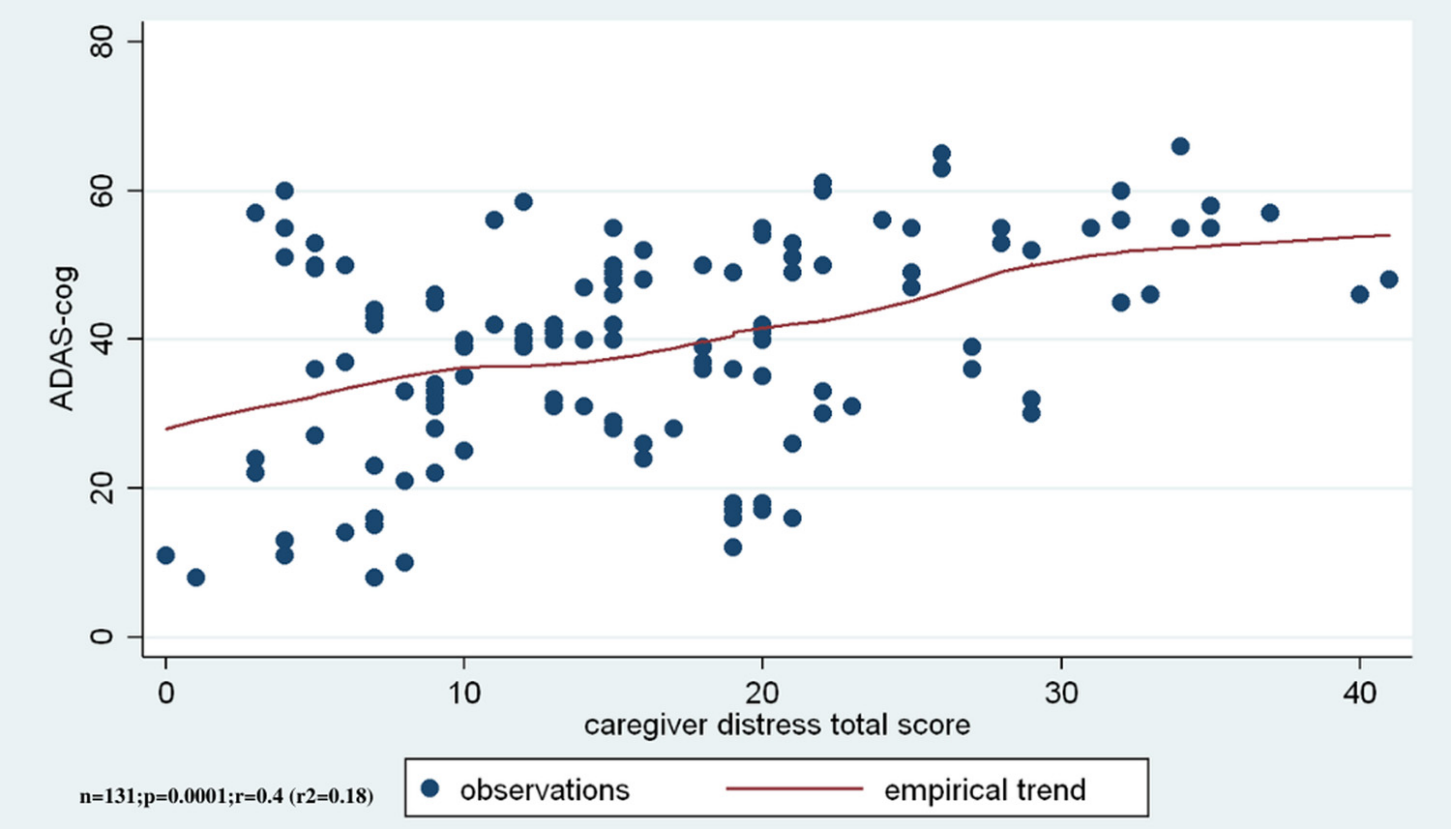
The impairment of cognitive functions correlate positively to the level of the caregiver’s distress.

The correlations between cognitive functions and NPS are discussed in this section. A moderate correlation was seen between the result of the test measuring cognitive functions and the total score of distress factor (*r* = 0.4, *r* ^2^ = 0.18, ANOVA *F*(1,129) = 28.5, *p* < 0.001), where greater impairment of cognitive functions was associated with higher levels of caregiver’s distress (Figure 6).

## 4 Discussion

This study focused on the examination of NPS in a Hungarian sample of patients with neurocognitive disorder, who had not received any dementia-related drug before. On average, the patient sample showed medium severity of major neurocognitive disorder (mean ADAS-Cog score: 39.4 points). Demographical data (gender distribution: 36.6% male, mean age: 77 years) of the sample were comparable to that of previously analysed samples in literature [19].

NPSs cause significant suffering and poor quality of life for both the patient and their caregivers [20]. It is generally agreed that every patient develops NPS. Even in the early phases of cognitive symptoms, NPS are estimated to appear in 35–85% of patients [21]. Their prevalence in a family setting is 56–98%; while in institutions, it is 91–96%. More than four symptoms seem to appear at the same time in 50% of patients [22]. In this research, 100% prevalence is shown. A possible explanation is that questions regarding symptoms, severity and frequency were individually asked and the caregiver did not think that the particular symptom, such as change in appetite, was related to the disease.

The findings of this investigation suggest that there is a significant correlation between quality of life, illness intrusiveness and NPSs. This, again, correlates with recent theories in the literature. However, few research have been made to measure the quality of life and illness intrusiveness of patients. In most of the cases, the quality of life and illness intrusiveness of the caregiver were the focus of the studies. Banerjee et al. [23] examined 101 patients with dementia and noted that the relationship between NPSs and quality of life is three times stronger than it is with cognitive symptoms.

NPSs have serious physical and mental impacts on the caregivers. A significant part of nursing time and distress is caused by NPS [24], and this is one of the main reasons for nursing home placement [25]. Nursing home placement significantly increases nursing costs [26]. Psychotic symptoms and disruptive behaviour like aggression and agitation are the most burdensome for the caregiver [27]. Regarding caregiver’s intrusiveness, the heaviest burdens in the analysed samples were motor disturbance, depression, agitation, changes in eating and appetite and delusion. These results are corroborated by several earlier research studies [28]. Similar results were obtained in our study.

The caregiver decompensates both in psychological and somatic aspects, their physical and mental health deteriorates and their problem-solving ability and strategy weakens [29]. The most frequent symptoms are fatigue, sleep disorder, changes in appetite, mood disorder and irritability [30]. These symptoms in themselves warrant treatment.

A British study published in 2006 also reported similar results and emphasised that the level of depression and anxiety strongly correlate with quality-of-life indexes [31]. Hurt et al. [32] examined 46 dementia patients and 116 caregivers with the aim to study the relationship between NPS and life quality. They concluded that NPSs have a negative relationship with quality of life and illness intrusiveness among patients and caregivers alike. In this study, it was established that the relationship between the burden of the relative and the NPSs was twice stronger than the relationship between the burden of the relatives and the cognitive functions.

The main principle of caring for dementia patients is to provide them with the feeling of safety and comfort, and the experience and joy of control over life situations while we endeavour to minimise different stress situations and maintain positive stimulating effects adjusted to the severity of dementia condition [33]. Experience has proven that a large part of stress situations could be eliminated if the caregiver had thorough knowledge about the course of the illness. The result derived from this investigation supports this as outlined by the trends. Another investigation [34] also verified that staying at home longer has a positive effect on patients’ prognosis and quality of life. The results of our research can also provide a useful basis for compiling future guidance for an action plan. This perhaps could be useful in composing a future guideline for an action plan. The caregivers’ education of the impact of NPSs as a cognitive symptom is an important part of this plan. Effective care for patients with neurocognitive disorders requires a multifaceted nursing approach with some different specialists [35,36,37]. This may include the physician, patient, family member/caregiver and other health care professionals. Perhaps they can all work as a team to ensure a better quality of life for patients. Therefore, NPSs need to be clarified, and the processes need to be thoroughly monitored.

The results obtained from this study were compiled in the light of some limitations. Conclusions must be drawn warily because this study was exploratory in nature. Additionally, it was a cross-sectional study; therefore, causative relationships could not be judged. Furthermore, the size of the sample in the examined group was not large enough, which prevents a firm conclusion.

It is also pivotal to note that there was no neuropathological validation of dementia subtypes. However, the use of samples from a naturalistic setting did provide a natural heterogeneity for the whole sample.

These observations have indeed shed some light on the general trends in patients with neurocognitive disorder regarding the examined associations. These noted trends may be very useful to clinicians in their everyday practice. This study also proves that future research needs to be carried out more specifically and rigorously in this direction.

## 5 Conclusion

This is the first systematic study which involves collecting information on NPS among Hungarian dementia patients in the presence of their relatives. The discoveries of this study, which examined a sample of dementia patients who had not received any dementia-related drug before, support previous findings from the literature concerning the high prevalence of NPS in dementia patients and the relevance of the NPS. This is also the first study in Hungary to draw attention to the impact of NPS on the burden of relatives as shown in the results. The objective is to open the mind of professionals to pay attention to NPS in addition to cognitive symptoms.

Although the results obtained are preliminary, future studies are essential for understanding these relationships in a clinical model of non-cognitive NPSs in neurocognitive disorders. Understanding this relationship could help physicians to establish diagnosis, inform and educate patients and their relatives about the likely outcome and course of the disease. The disease’s predictability can help patients, relatives and nurses to prepare for the long-term challenges both practically and psychologically. As a result of this, the home nursing option might be favoured significantly more and premature care home placement might eventually decrease.

The consistently reported high prevalence of NPS in patients with neurocognitive disorder, their connection to cognitive factors and their importance in the care together with this imperative finding draw emphasis to the clinical importance of this sub-syndrome in neurocognitive disorder that requires further study and clinical attention.

Again, this research is the first step to work out a complex nursing programme in which the caregiver plays an important role. The first stage was the survey of NPS in order to fully understand the correlations and overlaps between investigated groups of dementia and then to provide a better and more precise information for patients and their family members or caregivers. This will educate and prepare the caregivers about the symptoms to look out for as well as the challenges that might arise.

## Abbreviations

ADAS-Cog: Alzheimer’s Disease Assessment Scale-Cog
BPSD: Behavioural and psychological symptoms of dementia
IIRS: Illness intrusiveness rate scale
MMSE: Mini Mental State Examination
mNCD: Major neurocognitive disorder
NPI: Neuropsychiatric inventory
NPS: Neuropsychiatric symptoms
WBI-5: WHO well-being index short version
